# How coronavirus survives for hours in aerosols

**DOI:** 10.1063/5.0059908

**Published:** 2021-08-18

**Authors:** Sanghamitro Chatterjee, Janani Srree Murallidharan, Amit Agrawal, Rajneesh Bhardwaj

**Affiliations:** Department of Mechanical Engineering, Indian Institute of Technology Bombay, Mumbai 400076, India

## Abstract

COVID (CoronaVirus Disease)-19, caused by severe acute respiratory syndrome-CoronaVirus-2 (SARS-CoV-2) virus, predominantly transmits via airborne route, as highlighted by recent studies. Furthermore, recently published titer measurements of SARS-CoV-2 in aerosols have disclosed that the coronavirus can survive for hours. A consolidated knowledge on the physical mechanism and governing rules behind the significantly long survival of coronavirus in aerosols is lacking, which is the subject of the present investigation. We model the evaporation of aerosolized droplets of diameter ≤5 μm. The conventional diffusion-limited evaporation is not valid to model the evaporation of small size (*μ*m–nm) droplets since it predicts drying time on the order of milliseconds. Also, the sedimentation timescale of desiccated droplets is on the order of days and overpredicts the virus survival time; hence, it does not corroborate with the above-mentioned titer-decay timescale. We attribute the virus survival timescale to the fact that the drying of small (∼μm–nm) droplets is governed, in principle, by the excess internal pressure within the droplet, which stems from the disjoining pressure due to the cohesive intermolecular interaction between the liquid molecules and the Laplace-pressure. The model predictions for the temporal reduction in the aerosolized droplet number density agree well with the temporal decay of virus titer. The findings, therefore, provide insight on the survival of coronavirus in aerosols, which is particularly important to mitigate the spread of COVID-19 from indoors.

COVID (CoronaVirus Disease)-19 caused by Severe Acute Respiratory Syndrome-CoronaVirus-2 (SARS-CoV-2, referred to as coronavirus hereafter) has created huge health and an economic hazards throughout the world. The disease spreads via respiratory droplets.^1–3^ The fatality of the disease has engaged researchers in looking at the ways the disease spreads and the relative contributions between the different routes of the disease transmission.^4^ It was learnt that the main vector for the virus to attack a target cell is its rotational diffusivity, and that the triangularity of the coronavirus spike bulb decreases its rotational diffusivity.^5,6^ There are largely three different routes by which a susceptible person can attract infection: (a) direct exposure of a susceptible person to the respiratory droplets exhaled by an infected person, (b) inhalation of pathogen-containing aerosols suspended in air by a susceptible person, and (c) fomite transmission, i.e., if virus-laden droplets are deposited on a surface that serves as a secondary source of infection spread upon touch. The first two routes are called the airborne route of transmission.^7–10^ Usage of face masks and face shields is an effective measure to mitigate the disease spread.^11–15^ To minimize disease spread by fomite route, the effectiveness of surface disinfection,^16^ use of porous materials rather than impermeable materials,^17^ and antiviral surface-design have been studied.^18^

There is a number of recent evidence to believe that the COVID-19 disease is predominantly airborne.^19^ Respiratory droplets that transmit the virus from one subject to another have a varied size in a wide range (0.1 *μ*m–1 mm).^20–22^ The bigger droplets (typically ≥100 μm) can travel up to a certain distance before landing on a surface/on the ground due to gravity. For the case of bigger droplets, the infection is caused by direct exposure of a susceptible person to these droplets. A six-feet rule of social distancing is recommended to avoid disease transmission through large droplets.^23,24^ However, the case of disease transmission through the smaller-size droplets is distinct and requires special attention. The smaller droplets remain suspended in the air for a significant amount of time, making the air contaminated with pathogens. It is customary to consider that droplets of diameter ≤5 μm (also called droplet nuclei) remain suspended in the air as aerosols.^25,26^

Recently, the scientific community has taken a keen interest in analyzing the disease spread through aerosols.^27,28^ This is because a significant number of case studies revealed a number of COVID-19 positive cases at places where the social distancing rules or the fomite transmission precautionary measures were maintained.^19^ Understanding of the aerosol route of disease transmission becomes more crucial, especially in indoor environment with poor ventilation.^10,29–31^ The virus can remain suspended for a significant amount of time in the air of an indoor that was previously occupied by an infected person.^32^ Even bigger-size droplets may undergo evaporation before landing on a surface/ground and a small droplet-nuclei may be formed, which remains suspended in air, and the virus may still survive therein.^33^ Notably, previous virus titer measurements [dose $∼10^{5.25}$ 50% tissue-culture infectious dose (TCID_50_) per milliliter]^34^ disclosed that the coronavirus can sustain for hours in aerosols. It is, therefore, clear that the six-feet social distancing norm alone is not sufficient to curb disease spread via the airborne route. The survival timescale of the virus in aerosols must be accounted for to assess the total risk of airborne disease transmission. These facts highlight an urgent need to decipher the physical mechanism behind the survival of coronavirus in aerosols.

For an enveloped virus such as coronavirus, the aqueous phase of the respiratory droplet serves as the medium for survival of the virus, and therefore, the droplet-lifetime is correlated with the virus survival time, a fact that is well documented.^35,36^ The decay in the infectivity of 19 different viruses upon drying of virus-laden droplets on glass slides was experimentally investigated.^37^ Evaporation determines the eventual fate of the droplet, and therefore, the dynamics of droplet evaporation determines the virus viability contained within a respiratory droplet.^37,38^ It was demonstrated that the decay timescale of the virion concentration is correlated with the volume-decay of the respiratory droplet due to evaporation.^39^ Therefore, the infection spread and the virus survival are related to some extent to the ambient temperature and humidity.^1,40,41^ In our recent studies,^17,18,42^ we demonstrated that by considering a surrogate droplet of pure water, the drying timescale of the droplet and that of a residual thin-liquid film scale with the survival timescale of coronavirus on different surfaces. Therefore, the same idea can be extended further to analyze the drying of small droplets suspended in air in the context of coronavirus survival in aerosols.

Motivated by the aforesaid facts, herein, we investigate the evaporation dynamics of small pure water droplets (diameter 50 nm–5 μm) suspended in air and surrounded by water vapor. The governing mechanism of coronavirus survival in aerosols has not been explored yet, which is the subject of the present investigation. The motivation is further derived from our previous studies on the evaporation of respiratory droplets deposited on surfaces.^17,18,42^ In these studies, we found that while the bulk droplet undergoes a diffusion-limited evaporation and vanishes within seconds, after drying of the bulk-droplet, a residual thin-liquid film remains, whose evaporation is governed by the excess pressure within the thin film. The aforesaid excess pressure within the thin-film stems from the adhesive intermolecular interaction between the liquid and the solid molecules, which results in disjoining-pressure.^17,18,42^ Thereby, the drying of the residual thin-film is much slower, implying the coronavirus survives for hours/days on surfaces. Herein, we draw an analogy between the drying of the residual thin-film on surfaces to that of small (diameter 50 nm–5 μm) droplets suspended in air to model coronavirus survival in aerosols.

A schematic of the problem is shown in Fig. 1. Classically, the larger size droplets [diameter ≳10 μm; cf. Sec. S1(b) of the supplementary material] undergo a diffusion-limited evaporation.^43,44^ For smaller droplets of diameter ≤5 μm, which are typically the case of aerosols responsible for airborne disease transmission, the transport of energy and mass outside the droplet is considered ballistic,^45–47^ and the evaporation would be governed by the excess pressure within the droplet.^17,18,42^ To account for the excess pressure, the concept of disjoining pressure is extended herein to include cohesive intermolecular interaction within the small (∼μm–nm) droplets.^48,49^ In addition, the Laplace-pressure has to be taken into account because of the curvature of the liquid–vapor interface for the case of small (∼μm–nm) droplets.^50^ The disjoining-pressure due to cohesion and the Laplace pressure due to liquid–vapor interface curvature together determine the total excess internal pressure within the droplet.^50,51^ Based upon the above considerations, herein, we develop a model to look into the drying mechanism of small droplets of diameter ≤5 μm. As will be shown later, the drying of such small droplets is slower, and the drying timescale is well-correlated with the coronavirus survival timescale in aerosols found in earlier virus titer measurements.^34^ This way, the distinction of the present work is twofold: first, from a fundamental point of view, it imparts knowledge on the drying of suspended small (∼μm–nm) droplets. Second, from a COVID-19 point of view, it explains why the coronavirus survives for hours in aerosols? In this Letter, we use the term “droplet” rather than “droplet nuclei” to designate droplets of diameter ≤5 μm, which constitute aerosols.^25,26^

**FIG. 1.**
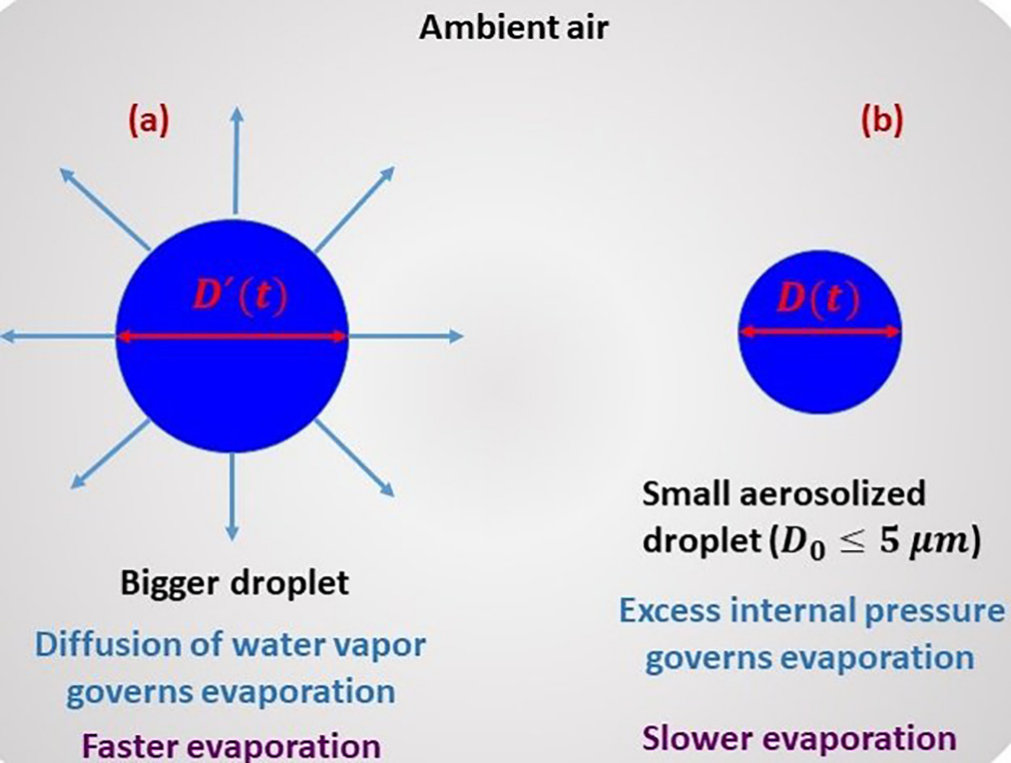
Schematic of the problem considered in the present work: (a) evaporation of a bigger droplet undergoing classical diffusion-limited evaporation and (b) evaporation of a small droplet of diameter ≤5 μm, whose evaporation is governed by the total internal excess pressure. The droplet in (b) represents an aerosolized droplet typically responsible for airborne disease transmission.

Next, we present different components of the model and the associated results. The model parameters have been chosen in a manner so as to enable us to feasibly compare the model with the earlier virus titer measurements in aerosols.^34^ Briefly, in the prior-art measurements, aerosols (≤5 μm) containing SARS-CoV-2 were generated with the use of a three-jet Collison nebulizer and fed into a Goldberg drum to create a homogeneous, well-dispersed aerosolized environment in a closed space. The samples were collected at 0, 30, 60, 120, and 180 min post-aerosolization for titer measurements. Consistent with the experimental conditions, the ambient temperature and relative humidity have been kept fixed at $T_{\mathrm{amb}}=21\;{}^{\circ}$C and H= 65%, respectively, in our present model. The aerosol droplets are assumed to be spherical in shape and having initial diameters *D*_0_ ∈ (50 nm, 5 *μ*m), an average diameter μ=2.5 μm, and a standard deviation σ=1.8 μm.^34,52–54^ The lower limit of *D*_0_ has been set 50 nm because the minimum diameter of SARS-CoV-2 virion is ∼50 nm.^55^ The assumption of homogeneous mixture of aerosolized droplets suspended in quiescent air has been widely used to model the evolution of pathogen in a closed space.^56^ The droplets once ejected are quickly dispersed in a large volume to reach a steady-state pathogen concentration. Hence, for a homogeneous dispersed mixture in a closed environment, each droplet has identical surroundings, and they are sufficiently distant from each other. The previous virus titer measurement, with which we aim to compare our model, also created a homogeneous aerosolized environment in a closed space (Goldberg drum). Hence, in accordance with the available knowledge, we seek to model the droplets as suspended and evaporating in still air, and they are isolated in terms of energy and mass transfer with their neighboring ones.

We assume quasi-steady evaporation for water droplets, justified as follows.^57^ The characteristic time for the liquid–vapor concentration to adjust to changes in the droplet shape is $t_{h}∼D_{0}^{2}/D_{\text{diff}}$, where $D_{\text{diff}}$ is the diffusivity of the liquid–vapor in air. The ratio of *t_h_* to the characteristic droplet evaporation time *t_f_* is $\phi =D_{0}^{2}/(t_{f}D_{\text{diff}})$ = $c_{\mathrm{sat}}(1-H)/\rho_{L}$, where *c_sat_* is the saturated liquid–vapor concentration in ambient air at *T_amb_*, and *ρ_L_* is the liquid density. Considering *H* = 0.65, *c_sat_* = 0.023 kg/m^3^, and *ρ_L_* = 1000 kg/m^3^ for water, the value of ϕ is estimated as $∼8\times 10^{-6}$ (ϕ≪1). Therefore, the quasi-steady evaporation is valid for water droplets. We note, however, that for evaporation of a highly volatile, low-boiling point liquid droplet (e.g., R134a) ϕ>1 limits the applicability of this assumption, and a two-way coupled heat and mass transfer between the evaporating droplet and the surrounding gas needs to be considered.^58^ Under these assumptions, we consider the evaporating droplets at the ambient temperature, and the temperature drop across the liquid-phase can be neglected for sufficiently small droplets (*μ*m–nm) with moderate thermal conductivity, and for simplicity, we assume the temperature of the liquid phase (*T_L_*) ≈ the liquid–vapor interfacial temperature (*T_lv_*) ≈ the saturation temperature (*T_sat_*) $\approx T_{\mathrm{amb}}$ [cf. Sec. S1(b) of the supplementary material].

Aerosols are commonly modeled by a lognormal distribution.^59,60^ According to the distribution function, the fraction of droplets between diameter *D*_0_ and $D_{0}+dD_{0}$ is expressed as

$$dF_{0}(D_{0})=\frac{1}{D_{0}\sigma \sqrt{2\pi }}\text{exp}\;\left(-\frac{{\left(\text{ln}\;(D_{0})-\mu \right)}^{2}}{2\sigma^{2}}\right)dD_{0}.$$ (1)

Notably, the distribution function is undefined as $D_{0}\to 0$, and thereby, it automatically excludes those particles, whose diameter (volume) tends to zero after complete evaporation. The probability density function (PDF) of $D_{0}\in$ (50 nm, 5 *μ*m) according to Eq. (1) is depicted in Fig. 2. This is the initial size distribution of the droplets which will evolve with time as the droplets evaporate and their sizes change.

**FIG. 2.**
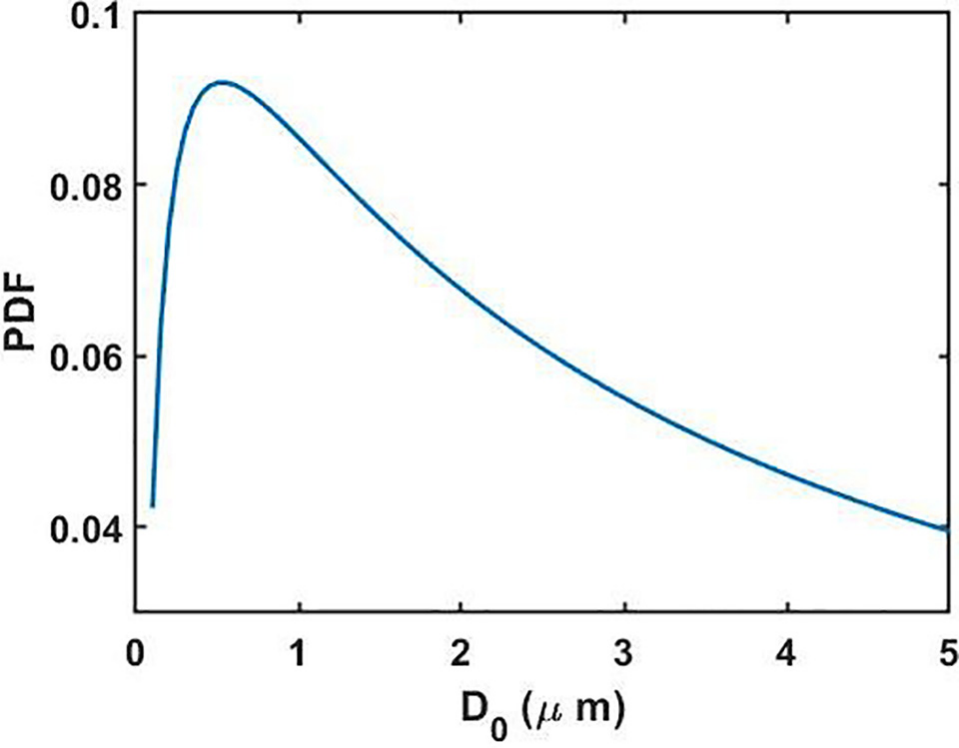
Probability density function (PDF) of the droplet initial diameter $D_{0}\in$ (50 nm, 5 *μ*m).

Next, we focus on the drying mechanism of droplets with $D_{0}\in$ (50 nm, 5 *μ*m). According to the classical diffusion-limited evaporation model, which is generally applied at large length-scales (∼mm),^43,44^ the droplet diameter $D^{\prime }(t)$ at time *t* is given by^43,44^

$$D^{\prime 2}(t)=D_{0}^{2}-\frac{8D_{\text{diff}}}{\rho_{L}}(c_{\mathrm{sat}}-Hc_{\mathrm{sat}})t,$$ (2)

where $D_{\text{diff}}$ of water–vapor in air at *T_amb_* is $2.6\times 10^{-5}$ m^2^/s; the other quantities and their values were defined earlier.

At small length-scales (∼nm–*μ*m), Eq. (2) does not hold as the mass and energy transport are treated as ballistic, and the kinetic approach needs to be adapted.^45–47^ Moreover, from Eq. (2), the diffusion-limited model predicts that a droplet with $D_{0}=5\;\mu$m takes *t *<4 ms to evaporate completely, while earlier virus titer measurements revealed that the titer decays to ∼85% of its initial value in a time of ∼3 h for the case of aerosols.^34^ Therefore, the diffusion-limited evaporation model is not appropriate to depict the droplet drying mechanism at small length-scales (50 nm, 5 *μ*m) and the associated coronavirus survival time in aerosols.

Previous studies have attributed longer survival of the virus in aerosols to the longer sedimentation timescale of the “desiccated” droplets.^61^ These desiccated droplets are formed when droplets with initial diameters *D*_0_ reach the “equilibrium” size defined by $D_{eq}=0.44\times D_{0}$ due to drying, after which they cease to evaporate further; this is attributable to the presence of solutes that are hygroscopic and retain some bound water.^62,63^ Despite the presence of solutes, it is plausible and a wide spread practice to model the evaporation and sedimentation of the droplets by including the properties of pure water (surface tension, density, and viscosity),^61–63^ as water constitutes ∼99% of the saliva content.^64^ The sedimentation dynamics of the desiccated droplets are elucidated in Sec. S2 of the supplementary material (cf. Fig. S3), wherein it is shown that $\forall D_{0}\in$ (1 *μ*m, 5 *μ*m); the sedimentation time *t_sed_* from an average height $z_{0}=2$ m varies within the range of 5–100 h. Clearly, *t_sed_* overpredicts the virus survival timescale (also evident from the droplet size distribution, cf. Fig. 2), and the observed 85% reduction in virus titer within 3 h (Ref. 34) cannot be explained by desiccated droplet sedimentation as well. Therefore, from the above discussion and in accordance with the available literature,^45–47^ herein, we seek to model the evaporation of aerosolized droplets with $D_{0}\in$ (50 nm, 5 *μ*m) under the ambit of the kinetic approach that is characterized by a slower evaporation rate.

In light of the knowledge gained from our previous studies,^17,18,42^ we attribute the longer survival of coronavirus in aerosols to the fact that the drying of droplets at small scales [$D_{0}\in$ (50 nm, 5 *μ*m)] is, in principle, governed by the excess internal pressure due to the cohesive intermolecular interaction within them. A detailed discussion on the supportive arguments along with the limits of applicability of the evaporation models is given in Sec. S1(b) of the supplementary material. The evaporative mass flux *j_evap_* (kg/m^2^ s) for the evaporation of a small droplet into its saturated vapor is described by the Hertz–Knudsen equation that uses the kinetic theory of gas,^46,48,49,65–70^

$$j_{\mathrm{evap}}=\frac{\rho_{V}}{\rho_{L}}\sqrt{\frac{1}{2\pi RT_{\mathrm{amb}}}}(p_{L}-p_{v}),$$ (3)

where *ρ_V_* = 0.023 kg/m^3^ is the concentration of water vapor at the ambient and R= 461.5 J/kg K is the specific gas constant of water vapor. Using these values, the prefactor outside the parenthesis of Eq. (7) has been computed as follows: $a=2.47\times 10^{-11}$ SI units, where $p_{L}-p_{v}$ represents the excess pressure within the droplet, which is stemmed from the disjoining-pressure (Π) due to the cohesive intermolecular interaction within the liquid and the Laplace-pressure (*p_c_*) due to the liquid–vapor interface curvature.^48,49^ For the droplet's instantaneous radius $R(t)=\frac{D(t)}{2}$, these two pressure terms can be written as^50,51^ follows. The disjoining pressure represents the difference between the pressure in the liquid phase (*p_L_*) and the normal component of the pressure tensor (*p_N_*)^51^

$$\Pi =p_{N}-p_{L}=-\frac{4A}{3\pi R^{3}(t)}.$$ (4)

The Laplace/capillary pressure represents the difference between the normal component of the pressure tensor (*p_N_*) and the pressure in homogeneous vapor phase (*p_v_*),^51^

$$p_{c}=p_{N}-p_{v}=\frac{2\gamma }{R(t)},$$ (5)

where $A=3.7\times 10^{-20}$ J and γ=0.072 J/m^2^ are Hamaker constant of cohesive interaction between water molecules and the surface tension of water, respectively.

From the conservation of mass

$$\rho_{L}\frac{dV(t)}{dt}=j_{\mathrm{evap}}.4\pi R^{2}(t),$$ (6)

where $V(t)=\frac{4}{3}\pi R^{3}(t)$ is the instantaneous droplet volume. Solving Eq. (6) using Eqs. (3)–(5), we obtain D(t)=2R(t) as a function of *t* as follows [see Sec. S1(a) of the supplementary material for the derivation]:

$$-\frac{\rho_{L}}{2\gamma a}\left[\frac{1}{8}(D^{2}(t)-D_{0}^{2})-\frac{2A}{6\pi \gamma }\text{log}\;\frac{16A+6\pi \gamma D^{2}(t)}{16A+6\pi \gamma D_{0}^{2}}\right]=t.$$ (7)

Equation (7) is solved iteratively with around time (*t*) and *R*_0_ to obtain the time variation of droplet diameter *D*(*t*) $\forall D_{0}\in$ (50 nm, 5 *μ*m). The model runs till each droplet reaches the equilibrium size defined by $D_{eq}=D(t)=0.44\times D_{0}$.^62,63^

First, we look into the relative contribution of the disjoining-pressure and the Laplace-pressure in governing the overall evaporation rate of the droplet. Figure 3 depicts that for droplets with a diameter greater than 2 μm, the Laplace pressure term is almost two orders of magnitude higher than the disjoining-pressure. The difference becomes more prominent at low droplet diameters. This trend can be understood from Eqs. (4) and (5): Π varies as $R^{-3}(t)$ and *p_c_* varies as $R^{-1}(t)$. Although *j_evap_* is dominated by the Laplace pressure, in our model, we have included the disjoining-pressure term for the sake of completeness and use Eq. (7) for further computations. A more detailed discussion on the relative contribution of Π and *p_c_* is given in Sec. S1(c) of the supplementary material.

**FIG. 3.**
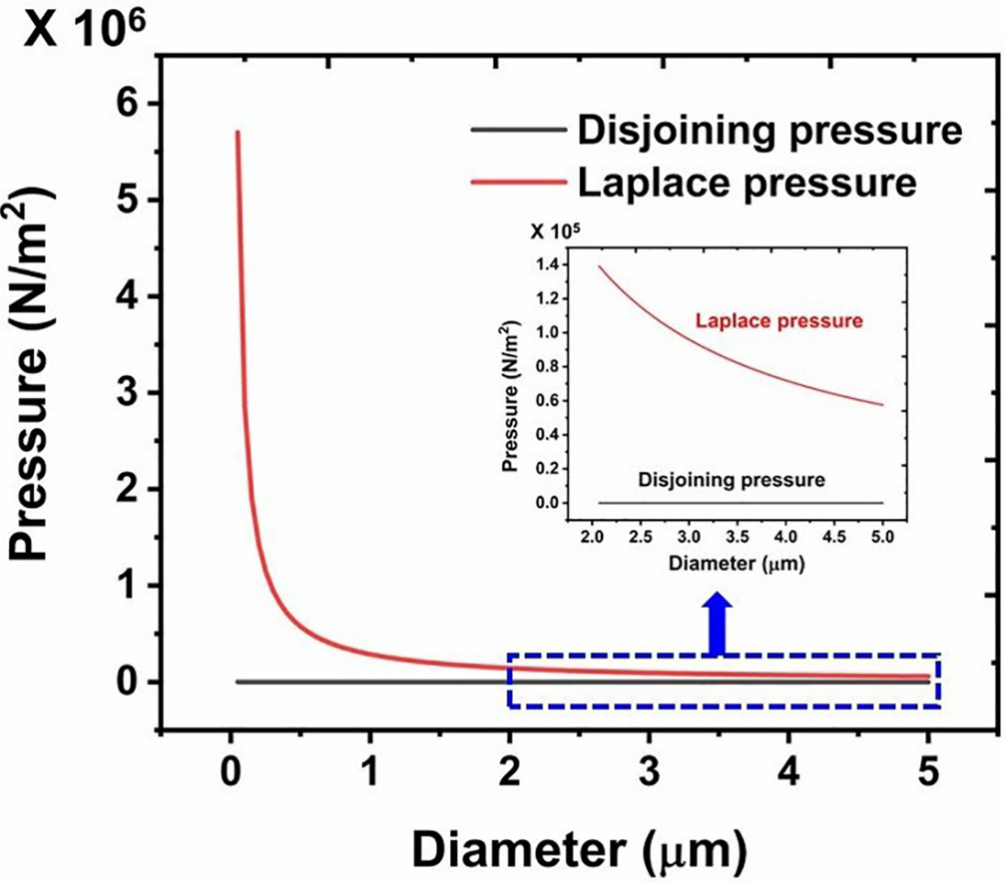
Variation of disjoining-pressure and Laplace pressure within the droplet for varying diameter.

Figure 4 shows the variation of *D*(*t*) with *t*, ∀ $D_{0}\in$ (50 nm, 5 *μ*m). The key features of the trends are as follows. The decay in *D*(*t*) with respect to *t* is linear for more than ∼80% of the droplet lifetime, and thereafter, a sharp decrease in the droplet diameter is observed. This may be attributed to the exponential increase in the Laplace pressure within the droplet for small diameter values (cf. Fig. 3). It is noteworthy that for the case of thin-liquid films on the solid surfaces, the disjoining pressure dominated the evaporation.^17,18,42^ In contrast, for the case of droplets with $D_{0}\in$ (50 nm, 5 *μ*m), the evaporation process is dominated by the Laplace-pressure. This feature marks the importance of the liquid–vapor interface curvature for the case of ∼μm–nm size droplets. Second, we note that the largest droplet with $D_{0}=5\;\mu$m takes ∼4 h to reach the equilibrium size by evaporation. This timescale is consistent with the decay timescale of virus titer found earlier, wherein a decay of 85% in the titer value was recorded in a timescale of ∼3 h.^34^ Previous studies reported evaporation timescale with similar order of magnitude for a ∼11.2 *μ*m diameter of water droplet.^46^ Hence, the present model that has been developed by considering the excess internal pressure within the droplet could capture the virus titer decay timescale with reasonable fidelity. Inspired by this finding, we further look into the details of total mass or droplet number density (number of droplets per unit volume of aerosol) with the flow of time and its correlation with the decay timescale of virus titer.

**FIG. 4.**
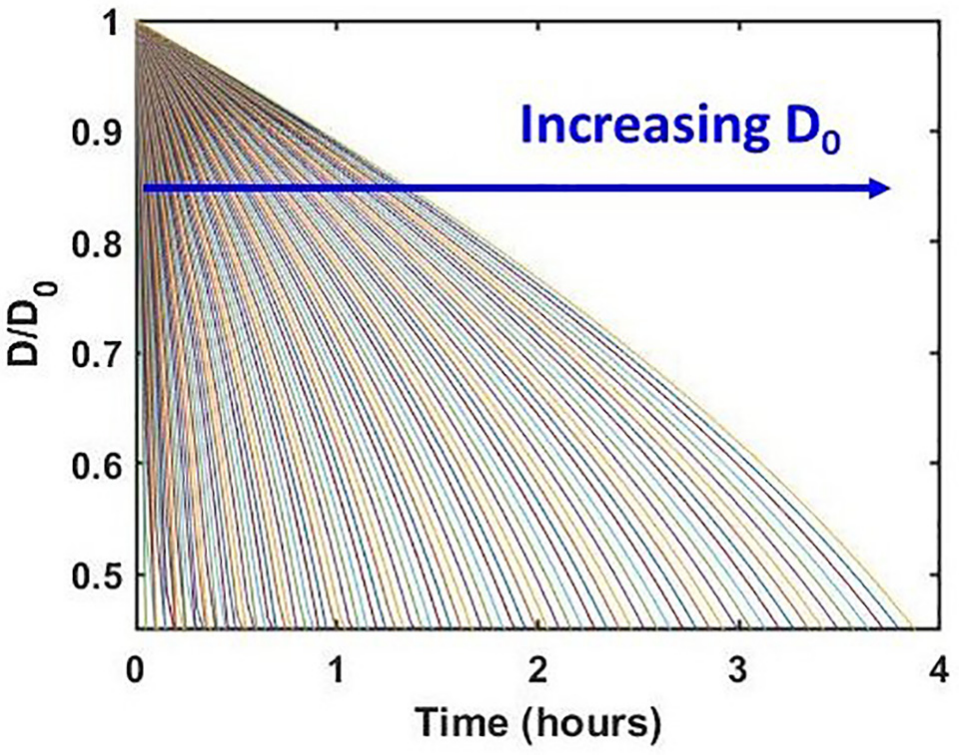
Variation of normalized droplet diameter [$D(t)/D_{0}$] with time ∀ $D_{0}\in$ (50 nm, 5 *μ*m) found from the model, where *D*_0_ is the initial diameter. The blue arrow indicates different cases of increasing *D*_0_ at a step of 49.5 nm.

To explore the loss in the total droplet mass/number density, we look into the evolution of the probability distribution function (PDF) with time. The PDF of *D*_0_, i.e., the initial particle size distribution, is depicted in Fig. 2. This distribution will evolve with time as the droplets evaporate following the governing law depicted in Eq. (7) (cf. Fig. 4). We designate the droplet diameters at time *t* as *D*(*t*), and the modified droplet diameters after time *t* + *dt* as $D_{\mathrm{new}}(t+dt)$. We assume that the droplets having diameters of *D*(*t*) with corresponding PDF *F*(*D*) at *t* have reduced to new diameters $D_{\mathrm{new}}(t+dt)$ at *t* + *dt* with corresponding new PDF $F_{\mathrm{new}}(D_{\mathrm{new}})$ after evaporation. Hence, if *dN_D_* represents the droplet number density between diameters *D*(*t*) and D(t)+dD(t), then by our assumption

$$dN_{D}=F_{\mathrm{new}}(D_{\mathrm{new}})dD_{\mathrm{new}}=F(D)dD.$$ (8)

From Eq. (8), the evolution of PDF is obtained as follows: (i) At time *t* = 0, the PDF of initial droplet diameters *D*_0_ is given by Eq. (1) and Fig. 2, which is considered as the initial condition. (ii) For obtaining the distribution function at later times, the product between the PDF at a diameter and the increment in the diameter at the (*i *+* *1)th time step is equated to the product between the PDF at a diameter and the increment in the diameter at the *i*th time step. This way, the new PDF at the (*i *+* *1)th time step is obtained from the older PDF at the *i*th time step, and the evolution of the probability distribution function with time can be computed. Droplets that reach the equilibrium size defined by $D(t)=0.44\times D_{0}$ are removed from the PDF.^62,63^

Figure 5 shows the evolution of PDFs at different times. The area under each curve represents the droplet number density at the corresponding time instant. From Fig. 5, the time-varying droplet number density has been obtained, which is shown in Fig. 6. We normalize the droplet number density at any time *t* with the initial number density (at *t *=* *0, cf. Fig. 2). Figure 6 shows the variation of normalized droplet number density with time. On the right axis, the published measurements^34^ of coronavirus titer at different time points in aerosols are plotted. It can be seen that there is reasonable qualitative agreement between the two datasets. The slope of the temporal virus titer decay matches well with the slope of the normalized droplet number density vs time curve. Hence, the time-varying virus titer scales with the time-varying mass of the aqueous phase of the aerosolized droplets. We recall from our earlier studies that for the case of respiratory droplets deposited on surfaces, the slope of decay of residual thin-film thickness matches qualitatively well with the temporal decay of coronavirus titer on a given surface.^17,18,42^ From Fig. 6, agreement between the time-variation of normalized droplet number density and the time-variation of virus titer in aerosols is consistent with our previous studies on virus survival on surfaces.^17,18,42^ Furthermore, in the earlier virus titer measurements in aerosols, a reduction in infectious titer from $10^{3.5}$ to $10^{2.7}$ (∼85%) TCID_50_ per liter of air was recorded in a time interval of ∼3 h.^34^ Our model captures this timescale for equivalent decay in the normalized number density with reasonable fidelity (cf. Fig. 6). The model predicts a timescale of ∼4 h for all the droplets in the aerosol cloud to reach the equilibrium size after evaporation. Therefore, the analytical model developed herein could capture the essential mechanism behind the long survival of coronavirus in aerosols with reasonable fidelity. The essential components of the model are that it considers the excess internal pressure as the main governing factor of evaporation of the aerosolized droplets along with an initial lognormal distribution in size, which consequently evolves in time as the droplets evaporate. Noteworthy that the temporal reduction of SARS-CoV-1 titer in aerosols also follows the same qualitative trend and decay timescale as that of SARS-CoV-2 under the same operating conditions.^34^ This fact expands the applicability of the present model; the temporal decay of aerosolized droplets' aqueous phase mass correlates with the temporal evolution of SARS-CoV-1 titer as well. All the model parameters chosen herein are consistent with the experiments^34^ against which the model is validated, as depicted in Fig. 6. The only exception is the lower limit of $D_{0}\in$ (50 nm, 5 *μ*m). To analyze the sensitivity of the model, we further vary the lower limit of *D*_0_ as 100, 500, and 1000 nm to compare the model predictions with the experiments. The results are shown in the supplementary material (cf. Sec. S3 and Fig. S4). All the curves generated by the model merge and agree with the titer measurements to the same extent. This is understood by realizing that the droplets with $D_{0}\geq 1\;\mu$m take longer to evaporate, irrespective of the lower limit of *D*_0_. They remain in the cloud to contribute to the distribution, and thereby the droplet number density. Therefore, the total lifetime of the droplet-cloud always corroborates with that of the measured virus survival timescale. Interestingly, for the case of thin-films on impermeable surfaces, the disjoining pressure effects are dominant,^42^ and thin-film lifetimes and the associated virus survival timescales were found to be ∼4–7 days; in aerosols, the Laplace-pressure is the main vector in determining the drying process, and the aqueous-phase lifetime along with the virus survival timescale is ∼3 h. Prima facie, this highlights the shorter lifetime of the virus in aerosols. Overall, the correlation between the lifetime of aqueous phase mass and virus survival is captured by the analysis.

**FIG. 5.**
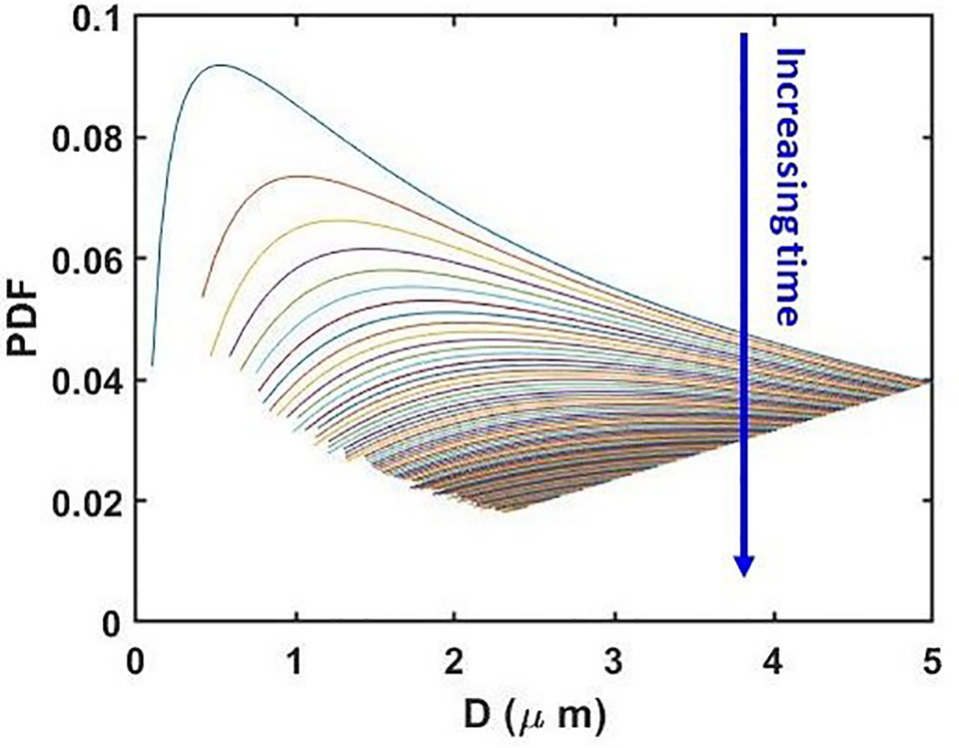
Temporal evolution of the probability density function of droplet diameters as the evaporation process goes on. The blue arrow indicates increasing time *t* from 0 to 5 h (18 000 s) at a step of 180 s.

**FIG. 6.**
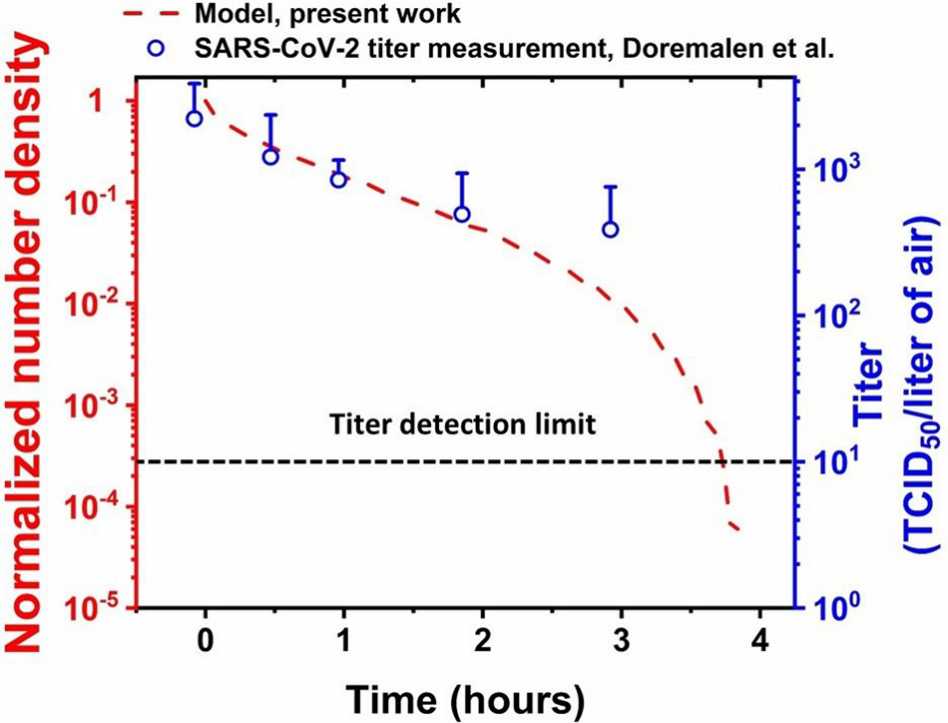
Time variation of normalized droplet number density derived from Fig. 5 (plotted as red dashed line). On the right axis, virus titer values at different times in aerosols (TCID_50_/liter of air) are plotted (open blue circles) as reported in the recent study,^34^ for comparison.

A few limitations of the model, which essentially stem from approximation of the respiratory droplets with surrogate droplets of pure water, were discussed in our previous papers.^17,18,42^ Nonetheless, the error associated with this approximation in the present model is within 25%.^71^ The effect of any internal convection and the associated shear stress on the virion particles are negligible for sessile droplets resting on surfaces.^20^ For the case of suspended droplet of spherical shape, these effects should be further negligible due to the homogeneity in the liquid–vapor interfacial temperature field.

In close, one of the contributing reasons behind the long survival time (∼hours) of coronavirus in aerosols has been deciphered herein. We have developed a semi-analytical model to understand the drying mechanism of aerosolized droplets (≤5 μm). The temporal decay of aerosolized droplet number density or total mass corroborates with the temporal decay of coronavirus titer reported in published measurements. The findings highlight the fact that coronavirus can survive for hours in aerosols in an indoor environment that was previously occupied by an infected individual. Therefore, the six-feet social distancing norm alone may not be sufficient to reduce the total risk of catching infection. The aforesaid survival timescale of coronavirus in aerosols must be kept in mind while occupying indoor spaces such as hospital rooms, railway/airport waiting halls, classrooms, and indoor sport stadiums.

## SUPPLEMENTARY MATERIAL

See the supplementary material for a detailed discussion on evaporation dynamics, and the relative contribution of different parameters, estimate of sedimentation times for droplets with different initial diameters, and the sensitivity analysis of the present model.

## Data Availability

The data that support the findings of this study are available from the corresponding author upon reasonable request.
